# Localization and quantification of intramuscular damage using statistical parametric mapping and skeletal muscle parcellation

**DOI:** 10.1038/srep18580

**Published:** 2015-12-22

**Authors:** Alexandre Fouré, Arnaud Le Troter, Maxime Guye, Jean-Pierre Mattei, David Bendahan, Julien Gondin

**Affiliations:** 1 Aix-Marseille Université, CNRS, CRMBM, UMR 7339, 13385, Marseille, France; 2 APHM, Hôpital de la Timone, CEMEREM, Pôle Imagerie Médicale, 13005, Marseille, France; 3 APHM, Hôpital de Sainte Marguerite, Service de Rhumatologie, Pôle Appareil Locomoteur, 13005, Marseille, France

## Abstract

In the present study, we proposed an original and robust methodology which combines the spatial normalization of skeletal muscle images, the statistical parametric mapping (SPM) analysis and the use of a specific parcellation in order to accurately localize and quantify the extent of skeletal muscle damage within the four heads of the *quadriceps femoris*. T_2_ maps of thigh muscles were characterized before, two (D2) and four (D4) days after 40 maximal isometric electrically-evoked contractions in 25 healthy young males. On the basis of SPM analysis of coregistrated T_2_ maps, the alterations were similarly detected at D2 and D4 in the superficial and distal regions of the *vastus medialis* (VM) whereas the proportion of altered muscle was higher in deep muscle regions of the *vastus lateralis* at D4 (deep: 35 ± 25%, superficial: 23 ± 15%) as compared to D2 (deep: 18 ± 13%, superficial: 17 ± 13%). The present methodology used for the first time on skeletal muscle would be of utmost interest to detect subtle intramuscular alterations not only for the diagnosis of muscular diseases but also for assessing the efficacy of potential therapeutic interventions and clinical treatment strategies.

Unaccustomed exercises and neuromuscular diseases can lead to the occurrence of skeletal muscle damage^1^,^2^,^3^,^4^,^5^,^6^,^7^. These alterations are associated with several physiological events leading to inflammatory processes within muscle^1^,^6^,^8^,^9^,^10^,^11^. Magnetic resonance imaging (MRI) appears as a method of choice to investigate *in vivo* the extent of muscle damage in healthy subjects^12^,^13^,^14^,^15^,^16^ or in patients with neuromuscular diseases^4^,^7^,^17^,^18^. MRI is a powerful non-invasive tool allowing for a spatially-resolved analysis of muscle tissue^4^,^15^,^19^. The increase in muscle proton transverse relaxation time (T_2_) has been identified as a relevant biomarker of muscle damage^15^,^19^,^20^,^21^,^22^ illustrating an inflammatory/edematous process^6^,^13^,^23^,^24^,^25^,^26^,^27^,^28^. Different T_2_ changes among muscles have been already reported after exercise-induced muscle damage^15^,^21^,^22^ and in dystrophic boys^4^. In most of these studies, the averaged T_2_ value was calculated in regions of interest within a given muscle thereby ignoring any spatial information. Although local T_2_ changes were assessed along muscles^19^,^22^, no study provided information on the accurate localization and extent of intramuscular damage into the three dimensions (3D) of a skeletal muscle.

Yet, accurate localization and quantification of muscle damage should provide more robust indices in diagnosis and longitudinal follow-ups of diseases or injuries. So far, only one study has reported information related to the distribution of intramuscular damage in dystrophic boys^7^. However, the analysis was only performed on a limited muscle volume (i.e., three MRI slices) and no information was provided on the localization of the most damaged areas within the muscle^7^. Moreover, we recently showed that neuromuscular electrostimulation (NMES) induced spatially heterogeneous T_2_ changes in *quadriceps femoris* (QF) muscle group^22^ with higher alterations in superficial muscles located beneath the stimulation electrodes (i.e., *vastus lateralis* [VL] and *vastus medialis* [VM]). On the sole basis of visual inspection of T_2_ maps, we consistently observed that altered muscle areas were heterogeneously distributed within the damaged muscle. Due to large inter-individual morphologic differences (e.g., muscle/tendon length, muscle volume), the 3D coregistration of MR images appears essential to accurately compare the localization of muscle alterations among individuals. Astonishingly, no study included this kind of image processing to characterize alterations within skeletal muscle.

To objectively localize and quantify the extent of tissues alterations, voxel-based analysis combining image coregistration and statistical parametric mapping (SPM) has been largely used for instance in the field of multiple sclerosis^29^,^30^,^31^ and Alzheimer disease^32^. Such a method allows analysis to be performed in a stereotactic space taking into account inter-individual morphology variability. Furthermore, specific parcellations have been used to discriminate several heart^33^,^34^ and brain^35^,^36^,^37^ areas and could be utilized to quantify the extent of local damage in individuals.

The data used in the present study were originally published using standard analyses techniques to compare localized multi-parametric MRI changes due to exercise-induced muscle damage among the four muscles of the QF^22^. In this paper, the aim was to localize and determine the spatial distribution of intramuscular damage using an original application of voxel-wise analysis on coregistered T_2_ maps of thigh skeletal muscles. In addition, we used a specific intramuscular parcellation in order to quantify the extent of the specific skeletal muscle alteration in deep and superficial areas.

## Results

### Distribution of muscle alterations

A significant increase in the VL and the VM muscles volume two days (D2) (+5.4 ± 3.7% and +4.2 ± 3.0% with p = 0.0001, respectively) and four days after (D4) (+8.5 ± 3.9% and +3.8 ± 3.2% with p = 0.0001, respectively) the NMES exercise were observed (Table 1) whereas no significant change occurred in the RF muscle (p > 0.05). A slight but significant increase in the *vastus intermedius* (VI) muscle volume was observed at D4 as compared to baseline (+2.2 ± 2.9% with p = 0.001, Table 1).

**Figure 1:**
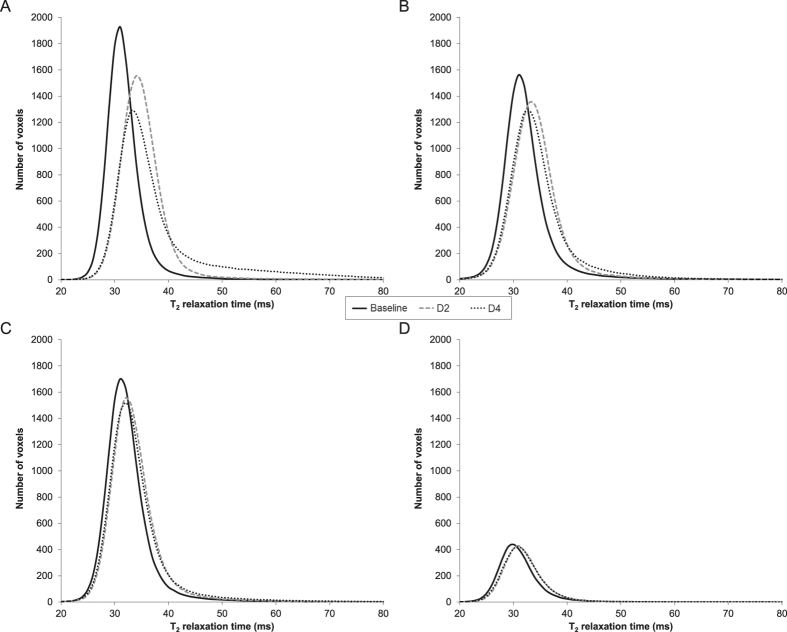
Distribution of intramuscular voxel-T_2_ values for the entire vastus lateralis (A), *vastus medialis* (B), *vastus intermedius* (C) and *rectus femoris* (D) muscles at baseline (solid black line), two days (dotted grey line) and four days (dotted black line) after NMES exercise. Error bars were removed for the sake of clarity.

For the VL and the VM, mean T_2_ values were significantly increased at D2 (+10.3 ± 5.5% and +5.7 ± 3.6%, respectively) and D4 (+19.5 ± 15.8% and +6.7 ± 5.0%, respectively) as compared to baseline (p = 0.0001, Table 1). As previously showed in damaged muscle of dystrophic boys^7^, a rightward shift in the relationships between the number of voxels and the voxel-T_2_ value was observed in both VL and VM muscles, reflecting an increased number of voxels with elevated T_2_ values (Fig. 1).

### Spatial normalization and statistical mapping analysis

**Figure 2:**
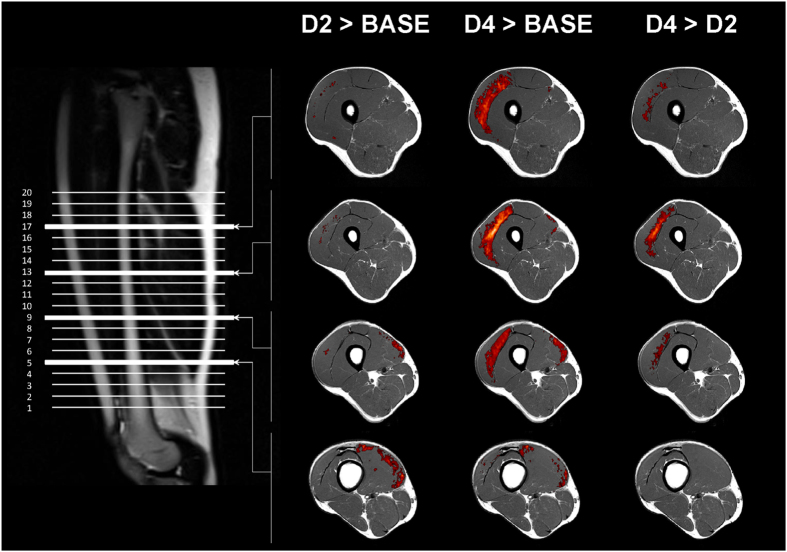
Statistical parametric mapping analysis for T_2_ increases in the *quadriceps femoris* between baseline (BASE) and acquisitions performed two (D2) and four (D4) days after the damaging exercise. Comparisons were performed with SPM8 software (one-way ANOVA, P < 0.001 and a cluster size > 100 voxels). The color scale (from red to yellow) represents the degree of significance. Results of the statistical analysis were overlaid on the T_1_-weighted axial images of the reference thigh used for spatial normalization on four levels on the muscle length.

The affine coregistration applied on images obtained at baseline, D2 and D4 for each subject and the non-linear coregistration of these images for the entire experimental population showed very high DICE similarity coefficients (DSCs of 0.89 ± 0.02 [range: 0.84–0.95] and 0.94 ± 0.02 [range: 0.90–0.96], respectively), thereby supporting the accuracy and robustness of a voxel-wise analysis. SPM analysis showed at D2 a significant increase in T_2_ in the center of the VL muscle whereas the VM was mainly altered in the superficial area of the distal slices at D2 (Fig. 2). A significant increase in T_2_ in the deep region over the full VL muscle length was further observed between D2 and D4.

### Localization of intramuscular alterations

**Figure 3:**
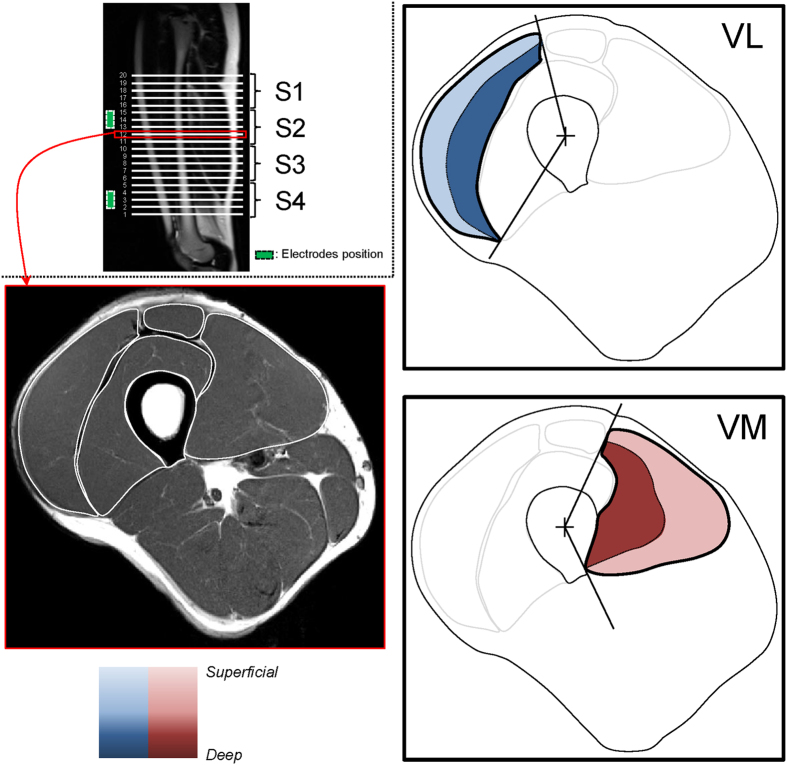
Schematic representation of the slices and electrodes positions on a sagittal image of the thigh. The axial slice from the S2 muscle parcel was used as an example for the specific parcellation of the *vastus lateralis* (VL) and the *vastus medialis* (VM) discriminating the superficial (light) and deep (dark) areas of each muscle.

Considering the results of the SPM analysis, the parcellation (Fig. 3) was only applied on the VL and the VM muscles. The proportion of altered voxels expressed in percentage of the total volume of each parcel was reported in Table 2 for the VL and in Table 3 for the VM. Considering the Gaussian distribution of the number of voxels with regard to the T_2_ values (Fig. 1), the relative alteration of the VL and the VM at baseline was 3.6 ± 0.8% in all the parcels of these two muscles. The absolute volume of alteration for the VL and the VM are available in the supplementary tables S1 and S2.

The VL analysis showed a significant depth × time interaction (p = 0.005) with larger alterations in both the deep and superficial regions at D2 (p = 0.004 and p = 0.007, respectively) and at D4 (p = 0.0001 and p = 0.0001, respectively) as compared to baseline. Muscle alterations were significantly larger in the deep regions at D4 as compared to the deep regions at D2 (p = 0.0001) and to the superficial regions at D4 (p = 0.0001). More specifically, on the basis of the depth × slice × time interaction (p = 0.0001), larger alterations were found in deep parcels as compared to the superficial ones of S1, S2 and S3 regions at D4 (p = 0.003). In addition, alterations were significantly larger in the deep regions at D4 in S1 and S2 (p = 0.02) as compared to S3 and S4 (Table 2).

The extent of alterations was lower in the VM as compared to the VL (e.g., 8% *vs.* 29% at D4). Indeed, there was no significant interaction among factors (p = 0.07 for depth × time interaction and p = 0.18 for slice × time interaction). However, a significant time effect was found (p = 0.001) with an increase in the relative volume of altered voxels within the VM at D2 (p = 0.03) and D4 (p = 0.0001) as compared to baseline illustrating that the relative alterations areas were homogeneously distributed within the VM muscle (Table 3). It is noteworthy that absolute volume of alteration extent in the VM reported in the supplementary tables S2 displayed significant difference between proximal/distal and superficial/deep parcels in accordance with the results of the SPM analysis (Fig. 2).

## Discussion

For the first time, we proposed an original methodology which combines the spatial normalization of skeletal muscle images, the SPM analysis and the use of a specific parcellation in order to accurately localize and quantify the extent of skeletal muscle damage within the four heads of the QF. The major results showed excellent T_2_ maps intra- and inter-individual coregistrations (DSCs > 0.84), a high sensitivity in the detection of muscle alterations by the SPM analysis and a relevant localization and quantification of intramuscular damage extent using the parcellation. This original method clearly illustrates that the altered VL muscle areas were mainly located in the deep and proximal regions whereas VM alterations were mainly found in the superficial and distal muscle zones. Additionally, a significant increase in the proportion of altered muscle voxels was detected in the deep regions of the VL between D2 and D4.

We previously reported heterogeneous alterations of the QF resulting from a single bout of NMES and the most affected muscles were located in direct contact with the stimulation electrodes^22^. Indeed, both VM and VL muscles were damaged and the extent of intramuscular alterations has been assumed to be different on the basis of a visual inspection of T_2_ maps^22^. In the present study, we showed that the intramuscular distribution of voxel-T_2_ values was modified in the VL and VM muscles at D2 and D4 with a rightward shift of the relationship between the number of voxels and voxel-T_2_ values. Interestingly, a similar shift has been previously described in damaged muscle of dystrophic boys as compared to healthy subjects^7^,^38^. It is noteworthy that the latter relationship became flatter at D4 for the VL describing a larger alteration of specific intramuscular areas. The global alteration of the VL and the VM in the present study (range: 6% – 19%) was not as large as what has been reported in dystrophic muscles (range: 30 – 40%). However, one has to keep in mind that T_2_ of fat tissue is higher than the skeletal muscle T_2_^39^. On that basis, it is likely that the large amount of intramuscular fat infiltration described in dystrophic muscle^4^,^40^ has influenced the T_2_ measurement^41^ in the study of Arpan *et al.*^7^. The potential T_2_ overestimation in skeletal muscle due to a partial fat volume effect further supports this explanation^42^.

In order to accurately localize the intramuscular alterations, we developed an original methodological approach combining the coregistration of skeletal muscle images and SPM analysis of T_2_ maps. Similar methodologies using voxel-wise statistical analyses have been largely used in brain alterations studies^29^,^30^,^32^ but this approach has never been used in the field of skeletal muscle alterations so far. In fact, the coregistration of skeletal muscle MR images remains challenging given the high variability in muscle shape and the relative position of muscles within the image. As a consequence, subtle difference in position among subjects and between MRI acquisitions (e.g., angular position of the leg, relative position of the coil on the thigh, stress of the coil on the thigh) can lead to a high inter- and intra- individual variability thereby making coregistration difficult. In the present study, we used ANTs algorithm^43^ in order to perform affine and non-linear 3D coregistrations of images in which each QF muscle was manually delineated. The T_2_ maps coregistrations were very robust with very high DICE similarity index. On that basis, SPM was used to localize significant T_2_ changes within QF muscle group on the entire experimental population. The corresponding statistical analysis allowed a more accurate detection of intramuscular alterations considering that no subjective visual assessments was used^44^ and that the inter-individual variability was taken into account from a large sample size. The voxel-wise analysis was combined with a specific parcellation in order to quantify the extent of local alterations of the damaged muscles in individuals. On that basis, two depth zones (i.e., superficial *vs.* deep) and four regions within the muscle length (i.e., from S1 [proximal part] to S4 [distal part]) were distinguished. Concerning the VL, we identified the larger alterations in the deep and proximal muscle areas.

We previously found that T_2_ changes associated to NMES-induced muscle damage were partly due to the edema – i.e., characterized by the change in the volume of VL, VM and VI^22^ -, the intramuscular membrane alterations – i.e., through the decrease in fractional anisotropy within the VL^22^ - and decreased intracellular resting pH of the whole QF^45^. Edema, inflammation processes and change in intracellular pH can lead to change in water properties within the muscle and then influence the T_2_ assessed by MRI^13^,^24^,^46^. On that basis, it is difficult to relate the local T_2_ change in the deep part of the VL to a single factor. However, the result of the present study concerning the localization of muscle alterations is an additional element in favor of the previously formulated hypothesis^22^ of a shear stress imposed by the friction between the VL and a less activated coplanar muscle (i.e., the VI) - leading to an unaccustomed strain within the VL during the NMES exercise. In a previous study, the T_2_ changes we quantified in each slice were not different among individuals^22^. Accordingly, we found no significant difference in the proportion of altered voxels among parcels S1, S2, S3 and S4 at D2 and D4 as compared to baseline when considering both deep and superficial regions together. However, the apparent similarity of the results between the two studies is rather associated to the relative homogeneity of knee extensors morphology across subjects (i.e., young healthy males) in our experimental population. It could be expected that results obtained would have been different in a population with a larger heterogeneity in morphologic characteristics (e.g., young/old; men/women). Using voxel-wise analysis and parcellation on the coregistrated T_2_ maps, we were able to accurately discriminate and quantify local muscle alterations in the deep and proximal parts of the VL. Then, on the contrary to what has been previously hypothesized regarding homogenous alterations along the full length of the VL^22^, we clearly showed using our approach that alterations were larger in the deep proximal parts of the muscle at D4 (i.e., at S1 and S2). Interestingly, this result illustrates a potential deleterious effect of the repeated activation of the same fibers beneath and close to the stimulation electrodes. In addition, the delayed increase in T_2_ between D2 and D4 detected in the deep and proximal areas using the combination of voxel-wise analysis and the parcellation could indicate a cumulative deleterious effect of the mechanical stress on muscle tissues.

Regarding the VM muscle, using the SPM analysis, we identified alterations in the superficial and distal areas. In addition, these alterations occurred in a larger muscle volume as compared to those we detected in the deep and proximal regions (Table S2). However, on the basis of the parcellation results, the proportion of altered muscle voxels was found to be homogeneous within the whole muscle. The low extent (i.e., ~6% at D2 and ~8% at D4) and the high inter-individual variability of muscle damage within the VM might explain the discrepancies between results obtained with the SPM analysis and the parcellation. Indeed, parcellation was performed on the T_2_ maps after an affine coregistration among the three tests for each subject whereas the SPM analysis was based on T_2_ maps after an additional non-linear coregistration across all the subjects. Then, the slight alterations detected in the superficial and distal regions of the VM were assumed to be due to the repeated activation of the same muscle fibers by the intramuscular nerve branches located beneath and close to the stimulation electrodes.

Our findings clearly demonstrated the sensitivity of our methodology to assess the changes in the location and extent of intramuscular alterations. From a methodological point of view, it appears essential to perform a spatial normalization of MR images - especially if variability of thigh morphology is large among individuals (e.g., femur length, position of muscle insertions, muscle/tendon length) – in order to compare the same part of the same muscle across subjects which was not necessarily the case in the previous studies assessing the localization of damage along the muscle^19^,^22^. The spatial normalization of MR images allowed the use of voxel-wise analysis which is a powerful method with a high sensitivity to detect the occurrence and the subtle time-dependent changes of skeletal muscle damage in a group of subjects.

In conclusion, the combined utilization of SPM analysis and the specific parcellation of skeletal muscle is a new robust statistical method allowing the detection, localization and quantification of intramuscular damage extent. Although T_2_ mapping of muscle damage does not provide information regarding the underlying mechanisms of injury or the cellular processes that are taking place, the robustness and originality of our methodological approach in the field of skeletal muscle physiology provides interesting perspectives for the longitudinal follow-up of skeletal muscle alterations in healthy subjects. Furthermore, the quantitative analysis of muscle alterations extent - i.e., relative and absolute volume of altered muscle - using the specific parcellation process is a method which could be of interest for the clinical follow up of tissue alterations in patients with neuromuscular diseases.

## Methods

These experiments were published with the same subjects in a previous paper^22^.

### Subjects

Twenty-five healthy men (22 ± 1 y, 178 ± 6 cm, 68 ± 7 kg) volunteered to participate in this study. None of them were engaged in any training or exercise programs. Subjects were instructed to avoid any intensive and non-familiar physical activities throughout the duration of the protocol. They were fully informed about the nature and the aim of the study and gave their informed written consent to participate in this study approved by the Local Human Research Ethics Committee Sud Mediterranée V (2012-04 A00449-34), and conducted in conformity with the Declaration of Helsinki. Subjects were asked to keep their diet habits and limit their alcohol consumption throughout the study period. They were instructed to avoid consuming caffeine and smoking before experimentations. Consumption of medication was prohibited during seven days before and five days after the NMES exercise. All testing sessions were performed at the same time of day. Three identical MRI sessions were performed six days before NMES exercise (baseline), at D2 and D4.

### NMES exercise

Subjects were seated on a chair (Multi-Form’, La Roque d’Anthéron, France) customized with a force sensor. Adjustable belts secured hip and ankle joints to hold the hip and knee angles at ~90° and ~100° respectively (0° corresponding to the joint fully extended). Both legs were stimulated simultaneously using three stimulation electrodes placed over the thigh, a 5 × 10 cm on the proximal part of the thigh (*i.e.*, placed ~5 cm below the inguinal ligament) and two 5 × 5 cm on the VL and VM muscle bellies. Biphasic symmetric rectangular pulses were delivered at a frequency of 100 Hz with a pulse duration of 400 μs (40 contractions, 5 s on and 35 s off throughout the NMES exercise) using a portable battery-powered stimulator (Compex^®^ Performance, DjoGlobal, France). Stimulation intensity was gradually increased in order to reach the highest tolerated (considering the pain threshold) level of evoked force for each subject as previously described^22^,^47^. This type of NMES protocol has been already shown to induce a prolonged maximal voluntary contraction force loss, delayed onset muscle soreness, increased plasma creatine kinase activity^45^,^47^ but more importantly heterogeneous T_2_ changes across the four heads of the QF^22^.

### MR images acquisition and post-processing

Subjects were positioned supine with the right leg centered in a 1.5-T super-conducting magnet (MAGNETOM Avanto, Siemens AG, Healthcare Sector, Erlangen, Germany). A flexible surface 6-channels coil (Siemens AG, Healthcare Sector, Erlangen, Germany) was placed around the right QF muscle. QF volume was determined from high-resolution T_1_-weighted images (20 slices, field of view (FOV) = 220 mm × 220 mm; matrix = 576 × 576; TR = 549 ms; TE = 13 ms; Number of repetitions (N_EX_) = 1; slice thickness = 6 mm; gap between slices = 6 mm, acquisition time = 5 min 18 s). T_2_-weighted images were acquired with a segmented (15 segments) echo planar imaging sequence with TE = 15, 25, 35, 45 and 55 ms. Other acquisition parameters were as follows: FOV = 220 mm × 220 mm; matrix = 192 × 192; TR = 4800 ms; N_EX_ = 1; number of slices = 20; slice thickness = 6 mm; gap between slices = 6 mm, Short-Tau Inversion-Recovery (STIR) for fat saturation; acquisition time = 5 min 10 s. The most distal slice was always acquired at approximately 20 mm (i.e., 5% of the thigh length measured for each subject) upper the proximal border of the patella. The stimulation electrodes were carefully localized by using oil capsules stuck on the skin surface and were observable on T_1_-weighted images (circular hyper-signals).

#### T_2_ mapping

Regions of interest (ROIs) were drawn with FSLView (FMRIB, Oxford, USA) in each slice by manually tracing the boundaries of the anatomic cross-sectional area of VL, VM, VI and *rectus femoris* [RF]. Using the truncated cone formula, QF muscle volume was calculated by summing the areas of all the slices, taking into account the slice thickness and the gaps between slices. T_2_ maps were generated by a linear fit on a pixel-by-pixel basis using the logarithm of the data to the equation (1):


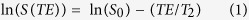


where S(TE) is the signal at time equal to TE and S_0_ is the equilibrium magnetization. Thereafter, T_2_ maps were resized to the T_1_-weighted images resolution. Regions of interest initially drawn on T_1_-weighted images were used to analyze T_2_ maps and to determine a mean T_2_ value for VL, VM, VI and RF. In addition, histograms representing the voxels numbers with respect to T_2_ relaxation times were determined for each muscle at baseline, D2 and D4.

#### Spatial normalization of the T_2_ maps

**Figure 4:**
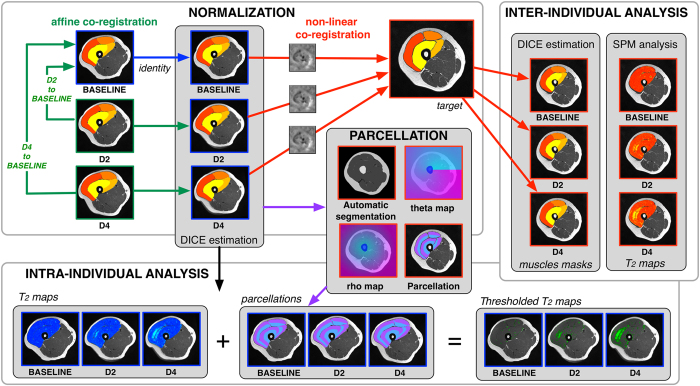
Pipeline of the analyses used for normalization of images with intra-subject affine co-registration and an additional inter-subject non-linear co-registration. The quality of the co-registrations was assessed using DICE similarity index. Muscle alterations were detected and localized two (D2) and four (D4) days after the damaging exercise using a statistical parametric analysis performed on the corresponding co-registered T_2_ maps from all the subjects. The extent of alterations was determined for each subject on T_2_ maps after the affine co-registration. A specific parcellation combining the automatic segmentation of the bone and polar coordinate system applied on the T_2_ maps allowed the detection of the altered muscle volume at D2 and D4 considering a threshold corresponding to the mean T_2_ increased of two standard deviations determined at baseline for each parcel.

As described in Fig. 4a, 3D affine coregistration was first performed using FLIRT algorithm of the FSL software (cost function = cross-correlation with 12 degrees of freedom) on the manually segmented masks of the four muscles at D2 and D4 to the manually segmented mask obtained at baseline. The affine transformation estimated by the intra-individual coregistration was then applied to the T_1_-weighted images and T_2_ maps. Thereafter, a 3D non-linear coregistration using ANTs library with symmetric diffeomorphic deformation model^43^ was performed for all the subjects. The parameters were optimized for large deformations of areas (with SyN option activated, cost function = cross-correlation, gradient step = 0.5, total-smoothing = 0.5, gradient-smoothing = 3, N-Time Steps = 1 and trunk = 256). The outputs were a set of warp and inverse warp deformation fields. The deformable field obtained from the inter-individual coregistration was then used to resample T_1_-weighted images and T_2_ maps (“normalization” module of the Fig. 4). Nearest-neighbor interpolation was applied to keep the integer values of the original labels. DSCs^48^ were used to estimate the overlap between manual segmentations of each muscle between the three tests after the coregistration processes for each subjects (intra-individual analysis) and between subjects (inter-individual analysis) using the equation (2).


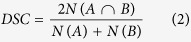


with *N*(*A*) the number of pixels in the structure *A*, *N*(*B*) the number of pixels in the structure *B*, 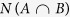
the number of common pixels between both structures. For individual coregistration, A represented the manual segmentation at baseline and B the manual segmentation at D2 or D4 after the 3D affine coregistration. For inter-individual coregistration, A represented the manual segmentation of the reference subject (i.e., defined as “target” in the Fig. 4) and B, the manual segmentations obtained for all the subjects after the 3D non-linear coregistration.

#### Statistical mapping analysis

Statistical parametric mapping software (SPM8, Wellcome Institute, London, UK) was used to compare the local T_2_ values across all the subjects at D2 and D4 on a pixel-to-pixel basis relative to similar data obtained in the same population at baseline. Analysis was performed for a repeated measurement for the 25 subjects considering cluster larger than 100 voxels^49^ and p = 0.001. The SPM analysis was used to detect alterations among and within each muscle of the QF (“inter-individual analysis” module of the Fig. 4).

#### Localization and quantification of altered muscle areas

First, a polar coordinate system was used to determine the position of each voxel of the T_2_ maps obtained after the affine co-registration (intra-individual coregistration). The origin of the localization system was determined as the bone gravity center on the basis of an automatic segmentation of bone, muscle and subcutaneous adipose tissue. The automatic segmentation has been developed in C++ using OpenCV library as previously published by Positano *et al.*^50^ in order to obtain a fast and fully automatic segmentation. While Positano *et al.* used a gradient vector flow snake algorithm^51^, we used a more classical polygonal active contour algorithm^52^ which determines the boundary between the subcutaneous fat and muscle tissues. The segmentation of bone was performed using the combination of a connected-component labeling analysis and mathematical morphology operations (i.e., a closing operation and filling region algorithm were applied successively on the largest component of voxels with the lowest mean intensity value into the muscle region).

Next to the automatic segmentation of the bone and localization of the bone gravity center, coordinates (*ρ: representing a distance from the bone gravity center*, *θ: representing an angular position*) were attributed to the voxels of the T_2_ maps and the associated manual muscle segmentation masks for three tests of each subject. Altered muscles were then automatically split into two depth levels – i.e., superficial and deep regions discriminating the half of the Δ*ρ* (between minimal and maximal *ρ* for each *θ* of each muscle and each slice). Thanks to the multi-slice acquisition, four groups of 5 slices were considered for analysis (from S1 to S4 corresponding to most proximal and distal zones, respectively). A schematic representation of the parcellation was displayed in Fig. 3 and its use in the raw data analysis was illustrated in the “parcellation” module of the Fig. 4. A threshold corresponding to the mean baseline value + two standard deviations for each parcel of each muscle (i.e., higher than the T_2_ value of 95% of the muscle voxels determined at baseline^7^ in each parcel) was applied on the T_2_ maps of each subject (“intra-individual analysis” module of the Fig. 4). The number of voxels (i.e., absolute and related to the muscle volume) with a T_2_ value higher than the threshold was quantified in each parcel of the thresholded T_2_ maps for each test and each subject. The stimulation electrodes were marked on slices 14 ± 2 for the VL and 3 ± 1 for the VM. Thus, the influence of electrodes position was mainly characterized in S1 and S2 for the VL and in S3 and S4 for the VM.

The normality of the data distribution was initially investigated using Shapiro-Wilk test. Two-way ANOVA (muscle × time) were used (Statistica, Statsoft, Tulsa, USA) to assess changes in volume and T_2_ of the whole muscles (i.e., VL, VM, VI and RF). SPM analysis was performed on T_2_ maps of each subject and for each time point (i.e., baseline, D2 and D4) after the coregistration processes (i.e., affine and non-linear). Multivariate ANOVAs were performed to assess the potential statistical difference in T_2_ changes according to time points and muscle parcels (deep/superficial and proximal/distal). A Tukey’s HSD post hoc analysis was used when appropriate.

The number of subjects was determined on the basis of a statistical power calculation (α = 0.05 and 1-β = 0.9) and previous measurements^47^ to detect a significant increase in the mean T_2_ of the whole muscle of 3.5%.

## Additional Information

**How to cite this article**: Fouré, A. *et al.* Localization and quantification of intramuscular damage using statistical parametric mapping and skeletal muscle parcellation. *Sci. Rep.* **5**, 18580; doi: 10.1038/srep18580 (2015).

## Supplementary Material

Supplementary Information

## Figures and Tables

**Table 1:** MRI parameters assessed before (Baseline), two days (D2) and four days (D4) after NMES-induced muscle damage (mean ± SD).

		Baseline	D2	D4
Muscle volume (cm^3^)	VL	544 ± 103	572 ± 102^a^	589 ± 108^a,b^
	VM	505 ± 78	525 ± 76^a^	524 ± 77^a^
	VI	569 ± 100	579 ± 96	581 ± 100^a^
	RF	168 ± 45	171 ± 43	172 ± 43
T_2_ (ms)	VL	32.5 ± 0.9	35.8 ± 1.9^a^	38.8 ± 5.0^a,b^
	VM	32.9 ± 0.8	34.8 ± 1.1^a^	35.1 ± 1.6^a^
	VI	33.1 ± 0.9	34.3 ± 1.0	34.5 ± 1.6
	RF	31.7 ± 0.9	32.7 ± 1.1	32.7 ± 1.3

VL: *vastus lateralis*; VM: *vastus medialis*; VI: *vastus intermedius*; RF: *rectus femoris*.

^a^significantly different from baseline (*P* < 0.001).

^b^significantly different from D2 (*P* < 0.001).

**Table 2:** Proportion of altered voxels in the VL muscle regions based on the T_2_ values distribution within each parcel at baseline, D2 and D4 (mean ± SD).

*% of altered voxels N* = *25*		S1	S2	S3	S4	All
BASELINE	Sup	3.5 ± 0.4	3.4 ± 0.3	3.2 ± 0.4	3.5 ± 0.9	**3.4** ± **0.3**
	Deep	3.2 ± 0.5	3.3 ± 0.5	3.7 ± 0.5	4.0 ± 0.8	**3.6** ± **0.4**
	All	**3.4** ± **0.3**	**3.3** ± **0.2**	**3.4** ± **0.3**	**3.8** ± **0.6**	**3.5** ± **0.3**
D2	Sup	12.8 ± 13.5^c^	17.8 ± 13.6	18.9 ± 14.6	20.2 ± 19.7^d^	**17.4** ± **13.2**^**a**^
	Deep	20.7 ± 15.5	20.3 ± 16.7	16.6 ± 15.1	15.7 ± 14.2	**18.3** ± **13.2**^**a**^
	All	**16.7** ± **11.9**	**19.0** ± **12.6**	**17.7** ± **12.4**	**18.0** ± **13.8**	**17.9** ± **10.7**^**a**^
D4	Sup	19.6 ± 13.6^c^	21.5 ± 12.3^c^	23.9 ± 16.3^**a,c**^	24.7 ± 29.6^**a**^	**23.3** ± **14.5**^a,c^
	Deep	42.2 ± 24.2^a,b^	41.5 ± 25.3^a,b^	32.1 ± 28.7^a,d,e^	28.3 ± 24.7^a,d,e,f^	**35.1** ± **24.9**^a,b^
	All	**30.9** ± **17.0**	**31.5** ± **17.0**	**28.0** ± **21.0**	**26.5** ± **24.8**	**29.2** ± **18.3**^a,b^

S1, S2, S3 and S4 represent the four groups of five slices of the muscle (S1 corresponding to the most proximal part of the muscle). Sup: superficial muscle region, Deep: deep muscle region.

^a^significantly different from baseline (*p* ≤ 0.02).

^b^significantly different from D2 (*p* ≤ 0.02).

^c^significantly different from deep (*p* ≤ 0.01).

^d^significantly different from S1 (*p* ≤ 0.02).

^e^significantly different from S2 (*p* = 0.02).

^f^significantly different from S3 (*p* ≤ 0.02).

**Table 3:** Proportion of altered voxels in the VM muscle regions based on the T_2_ values distribution within each parcel at baseline, D2 and D4 (mean ± SD).

*% of altered voxels N* = *25*		S1	S2	S3	S4	All
BASELINE	Sup	3.6 ± 0.7	3.9 ± 0.5	3.9 ± 0.6	3.7 ± 0.6	**3.8** ± **0.3**
	Deep	3.9 ± 0.7	3.6 ± 0.5	3.5 ± 0.4	4.1 ± 0.7	**3.8** ± **0.4**
	All	**3.7** ± **0.5**	**3.7** ± **0.4**	**3.7** ± **0.4**	**3.9** ± **0.4**	**3.8** ± **0.3**
D2	Sup	5.8 ± 3.2	7.2 ± 8.9	6.6 ± 3.9	8.9 ± 7.9	**7.1** ± **4.7**
	Deep	4.1 ± 2.4	4.7 ± 2.6	5.5 ± 3.7	5.8 ± 3.2	**5.0** ± **2.3**
	All	**5.0** ± **2.3**	**6.0** ± **5.3**	**6.0** ± **3.3**	**7.3** ± **5.0**	**6.1** ± **3.2**^**a**^
D4	Sup	7.5 ± 5.0	9.8 ± 11.3	10.4 ± 8.7	8.1 ± 5.1	**9.3** ± **6.9**
	Deep	5.0 ± 2.3	6.2 ± 4.7	8.1 ± 6.1	9.6 ± 9.4	**6.9** ± **3.6**
	All	**6.2** ± **3.1**	**8.0** ± **7.2**	**9.3** ± **6.5**	**8.9** ± **6.0**	**8.1** ± **4.6**^**a**^

S1, S2, S3 and S4 represent the four groups of five slices of the muscle (S1 corresponding to the most proximal part of the muscle). Sup: superficial muscle region, Deep: deep muscle region.

^a^significantly different from baseline (*p* ≤ 0.04).
