# Supplementary material for: Localization and quantification of intramuscular damage using statistical parametric mapping and skeletal muscle parcellation
Source: Sci Rep. 2015 Dec 22;5:18580. doi: 10.1038/srep18580 (PMC4686971; doi:10.1038/srep18580)
Supplement: Supplementary Information [file srep18580-s1.doc]

**Supplementary materials**

**Localization and quantification of intramuscular damage using statistical parametric mapping and skeletal muscle parcellation**

Alexandre Fouré^1^, Arnaud Le Troter^1^, Maxime Guye^1,2^, Jean-Pierre Mattei^1,3^, David Bendahan^1^, Julien Gondin^1^

^1^Aix-Marseille Université, CNRS, CRMBM, UMR 7339, 13385, Marseille, FRANCE.

^2^APHM, Hôpital de *la Timone*, CEMEREM, Pôle d’imagerie médicale, 13005, Marseille, FRANCE.

^3^APHM, Hôpital de *Sainte Marguerite*, Service de Rhumatologie, Pôle Appareil Locomoteur, 13005, Marseille, FRANCE.

**Table S1: Absolute altered volume of the VL muscle regions based on the T_2_ values distribution within each parcel at baseline, D2 and D4 (mean ± SD).**

| ***Volume of alteration (cm^3^)***  ***N = 25*** | | **S1** | **S2** | **S3** | **S4** | **All** |
| --- | --- | --- | --- | --- | --- | --- |
| **BASELINE** | **Sup** | 11 ± 2 | 11 ± 2 | 7 ± 1 | 2 ± 1 | **30 ± 6** |
|  | **Deep** | 8 ± 2 | 9 ± 2 | 7 ± 2 | 2 ± 1 | **26 ± 6** |
|  | **All** | **19 ± 3** | **20 ± 4** | **14 ± 3** | **4 ± 2** | **56 ± 11** |
| **D2** | **Sup** | 42 ± 37 | 59 ± 37^a^ | 39 ± 26 | 7 ± 7 | **149 ± 94^a^** |
|  | **Deep** | 56 ± 39^a^ | 55 ± 47^a^ | 30 ± 29 | 9 ± 9 | **147 ± 111^a^** |
|  | **All** | **98 ± 61^a^** | **113 ± 70^a^** | **69 ± 45^e^** | **16 ± 13^d,e,f^** | **296 ± 166^a^** |
| **D4** | **Sup** | 69 ± 52^a,c^ | 75 ± 38^a,c^ | 53 ± 35^a,d,e^ | 15 ± 16^d,e,f^ | **212 ± 126^a,c^** |
|  | **Deep** | 117 ± 72^a,b^ | 117 ± 78^a,b^ | 59 ± 55^a,d,e^ | 10 ± 12^d,e,f^ | **303 ± 204^a,b^** |
|  | **All** | **186 ± 114^a,b^** | **192 ± 109^a,b^** | **111 ± 87^a,d,e^** | **25 ± 26^d,e,f^** | **515 ± 312^a,b^** |

*S1, S2, S3 and S4 represent the four groups of five slices of the muscle (S1 corresponding to the most proximal part of the muscle). Sup: superficial muscle region, Deep: deep muscle region.*

*a: significantly different from baseline (P ≤ 0.02), b: significantly different from D2 (P ≤ 0.02), c: significantly different from deep (P ≤ 0.01), d: significantly different from S1 (P ≤ 0.02), e: significantly different from S2 (P ≤ 0.02), f: significantly different from S3 (P ≤ 0.02).*

**Table S2: Absolute altered volume of the VM muscle regions based on the T_2_ values distribution within each parcel at baseline, D2 and D4 (mean ± SD).**

| ***Volume of alteration (cm^3^)***  ***N = 25*** | | **S1** | **S2** | **S3** | **S4** | **All** |
| --- | --- | --- | --- | --- | --- | --- |
| **BASELINE** | **Sup** | 4 ± 1 | 9 ± 2 | 13 ± 3 | 11 ± 3 | **37 ± 8** |
|  | **Deep** | 3 ± 1 | 5 ± 1 | 8 ± 2 | 8 ± 2 | **23 ± 5** |
|  | **All** | **7 ± 2** | **14 ± 3** | **21 ± 4^d^** | **18 ± 5** | **61 ± 12** |
| **D2** | **Sup** | 8 ± 4 | 17 ± 17 | 22 ± 12 | 24 ± 18 | **71 ± 42^a,c^** |
|  | **Deep** | 3 ± 2 | 7 ± 4 | 12 ± 6 | 11 ± 6 | **33 ± 14** |
|  | **All** | **11 ± 5** | **24 ± 18^d^** | **33 ± 16^d^** | **35 ± 22^d^** | **104 ± 50^a^** |
| **D4** | **Sup** | 13 ± 10 | 25 ± 24 | 35 ± 28 | 26 ± 22 | **99 ± 68^a,c^** |
|  | **Deep** | 4 ± 1 | 10 ± 11 | 18 ± 15 | 16 ± 11 | **47 ± 33** |
|  | **All** | **16 ± 11** | **35 ± 29^a,d^** | **53 ± 37^a,b,d,e^** | **42 ± 28^a,d^** | **146 ± 90^a,b^** |

*S1, S2, S3 and S4 represent the four groups of five slices of the muscle (S1 corresponding to the most proximal part of the muscle). Sup: superficial muscle region, Deep: deep muscle region.*

*a: significantly different from baseline (P ≤ 0.02), b: significantly different from D2 (P ≤ 0.02), c: significantly different from deep (P ≤ 0.01), d: significantly different from S1 (P ≤ 0.02), e: significantly different from S2 (P < 0.01).*
